# Phosphatidylinositol 3-Kinase-Associated Protein (PI3KAP)/XB130 Crosslinks Actin Filaments through Its Actin Binding and Multimerization Properties *In Vitro* and Enhances Endocytosis in HEK293 Cells

**DOI:** 10.3389/fendo.2016.00089

**Published:** 2016-07-11

**Authors:** Daisuke Yamanaka, Takeshi Akama, Kazuhiro Chida, Shiro Minami, Koichi Ito, Fumihiko Hakuno, Shin-Ichiro Takahashi

**Affiliations:** ^1^Laboratory of Cell Regulation, Department of Animal Resource Sciences, Graduate School of Agriculture and Life Science, The University of Tokyo, Bunkyo-ku, Japan; ^2^Laboratory of Food and Physiological Models, Department of Veterinary Medical Sciences, Graduate School of Agriculture and Life Science, The University of Tokyo, Kasama, Japan; ^3^Department of Bioregulation, Nippon Medical School, Kawasaki, Japan

**Keywords:** actin, adaptor protein, phosphatidylinositol 3-kinase, protein self-assembly, endocytosis

## Abstract

Actin-crosslinking proteins control actin filament networks and bundles and contribute to various cellular functions including regulation of cell migration, cell morphology, and endocytosis. Phosphatidylinositol 3-kinase-associated protein (PI3KAP)/XB130 has been reported to be localized to actin filaments (F-actin) and required for cell migration in thyroid carcinoma cells. Here, we show a role for PI3KAP/XB130 as an actin-crosslinking protein. First, we found that the carboxyl terminal region of PI3KAP/XB130 containing amino acid residues 830–840 was required and sufficient for localization to F-actin in NIH3T3 cells, and this region is directly bound to F-actin *in vitro*. Moreover, actin-crosslinking assay revealed that recombinant PI3KAP/XB130 crosslinked F-actin. In general, actin-crosslinking proteins often multimerize to assemble multiple actin-binding sites. We then investigated whether PI3KAP/XB130 could form a multimer. Blue native-PAGE analysis showed that recombinant PI3KAP/XB130 was detected at 250–1200 kDa although the molecular mass was approximately 125 kDa, suggesting that PI3KAP/XB130 formed multimers. Furthermore, we found that the amino terminal 40 amino acids were required for this multimerization by co-immunoprecipitation assay in HEK293T cells. Deletion mutants of PI3KAP/XB130 lacking the actin-binding region or the multimerizing region did not crosslink actin filaments, indicating that actin binding and multimerization of PI3KAP/XB130 were necessary to crosslink F-actin. Finally, we examined roles of PI3KAP/XB130 on endocytosis, an actin-related biological process. Overexpression of PI3KAP/XB130 enhanced dextran uptake in HEK 293 cells. However, most of the cells transfected with the deletion mutant lacking the actin-binding region incorporated dextran to a similar extent as control cells. Taken together, these results demonstrate that PI3KAP/XB130 crosslinks F-actin through both its actin-binding region and multimerizing region and plays an important role in endocytosis.

## Introduction

Actin is a highly conserved essential component of the cytoskeleton that generates forces to drive cell motility, cell division, and cell adhesion, maintains cell morphology and polarity, and functions in endocytosis and vesicular trafficking (1). These processes are indispensable for a variety of biological processes such as development of endocrine tissues, maintenance of cell functions, synthesis and secretion of hormones in endocrine cells (2–4), and macropinocytosis and metastasis in cancer cells (5, 6). Actin filaments are constantly remodeled through polymerization of actin monomers into filaments and their subsequent organization into functional higher-order structures. The remodeling of the actin cytoskeleton is regulated by more than 100 different actin-binding proteins, and actin-crosslinking proteins regulate the organization of actin filament networks and bundles and contribute to the development of specialized cellular structures including filopodia, lamellipodia, and stress fibers (7).

Actin filament-associated protein (AFAP) family of proteins consists of AFAP-110, AFAP1-like1 (AFAP1L1), and phosphatidylinositol 3-kinase-associated protein (PI3KAP)/XB130 (8–12). Among AFAP family members, AFAP-110 was first reported to bind to and crosslink actin filaments (13, 14). By these activities, AFAP-110 modulates changes in actin filament integrity (15). The actin-binding domain (ABD) of AFAP-110 is conserved in AFAP1L1, which has also been reported to be localized to the actin-based structure, invadosome (10). In contrast to these two AFAP family members, the ABD is not conserved in PI3KAP/XB130. Nevertheless, it has been reported that PI3KAP/XB130 is localized to cortical actin structures including lamellipodia (16, 17). These results suggest that PI3KAP/XB130 controls organization of actin filaments through novel mechanisms distinct from other AFAP family members.

We have been studying molecular mechanisms for the signaling crosstalk between insulin-like growth factors (IGF) and tropic hormones in endocrine cells. In the course of our study, we found that expression of PI3KAP/XB130 was induced in response to thyroid-stimulating hormone (TSH) in the thyroid cells and in the thyroid gland, this increase was required for signaling crosstalk with IGF leading to synergistic cell proliferation (12). Similar synergistic effects of IGF and tropic hormones are observed in various other endocrine tissues, such as the adrenal gland, testis, and ovary, and in these tissues, the signaling crosstalk is indispensable for the cells to proliferate, differentiate, and maintain their functions (18–22). By contrast, expression of PI3KAP/XB130 has been reported in endocrine cancers such as thyroid cancer cells, and PI3KAP/XB130 knockdown inhibits cell proliferation, survival, migration, and invasion in these cell types (16, 23–25). These findings indicate a possibility that PI3KAP/XB130 plays important roles in endocrine tissues and endocrine cancers. PI3KAP/XB130 is considered to function as an adaptor protein, based on the molecular structures that can interact with other molecules. These structures include two PH domains, a coiled-coil domain and several binding motifs recognized by SH2 or SH3 domains. Indeed, PI3KAP/XB130 is tyrosine-phosphorylated by activation of cAMP pathways or by overexpression of c-Src or RET/PTC tyrosine kinase and thereby interacts with PI 3-kinase, which is likely to mediate signal transduction for cell proliferation (11, 12, 23–27). However, it remains unclear, so far, whether PI3KAP/XB130 interacts with actin itself or actin-binding proteins.

This study was undertaken to elucidate molecular mechanisms by which PI3KAP/XB130 regulates the organization of actin filaments. In this study, we demonstrate that PI3KAP/XB130 binds to actin filaments *via* a novel actin-binding region different from the ABD of AFAP-110 or AFAP1L1. Moreover, we also show that PI3KAP/XB130 forms multimers, and multimerization of this protein can lead to crosslinking of actin filaments. To uncover a novel function of this protein in an actin-related process, we further verified whether PI3KAP/XB130 is involved in regulation of endocytosis in HEK 293 cells. Here, we propose a novel role for PI3KAP/XB130 as an actin-crosslinking protein, and this may link actin localization of PI3KAP/XB130 and regulation of endocytosis mediated by this protein.

## Materials and Methods

### Materials

Dulbecco’s Modified Eagle’s medium (DMEM), phosphate-buffered saline (PBS), and Hanks’ balanced salt solution were obtained from Nissui (Tokyo, Japan). Fetal bovine serum (FBS) and calf serum (CS) were obtained from JRH Bioscience (Tokyo, Japan). Anti-Myc monoclonal antibody (9E10) was purchased from Millipore (Billerica, MA, USA). Anti-FLAG M2 antibody and anti-α-tubulin antibody (B-5-1-2) were obtained from Sigma-Aldrich (St. Louis, MO, USA). Anti-GFP monoclonal antibody (B-2) was purchased from Santa Cruz Biotechnology (Santa Cruz, CA, USA). Anti-PI3KAP/XB130 antibody was raised in our laboratory as previously described (12). Alexa Fluor 488-conjugated anti-mouse IgG antibody was from Invitrogen (Carlsbad, CA, USA). Horseradish peroxidase (HRP)-linked anti-mouse IgG antibody and HRP-linked anti-rabbit IgG antibody were purchased from GE Healthcare (Buckinghamshire, UK). Other chemicals were of reagent grade available commercially.

### Cell Culture

NIH3T3 cells were purchased from Health Science Research Resources Bank (Osaka, Japan). HEK293T cells were a kind gift from Dr. Kunio Shiota (The University of Tokyo, Tokyo, Japan). HEK293 cells were kindly provided by Dr. Koichi Suzuki (Teikyo University, Tokyo, Japan). NIH3T3 cells, HEK293T cells, and HEK293 cells were cultured in DMEM containing 1 mg/ml NaHCO_3_, 50 IU/ml penicillin, 50 μg/ml streptomycin, 0.5 μg/ml amphotericin B, and 100 μg/ml kanamycin supplemented with 10% FBS (NIH3T3 cells and HEK293 cells) or 10% CS (HEK293T cells). FRTL-5 rat thyroid follicular cells (28) were kindly provided by the late Dr. Leonard Kohn (Ohio University and Edison Biotechnology Institute, Athens, OH, USA). FRTL-5 cells were cultured as previously described (12).

### Plasmid Construction

The mammalian expression plasmid pShuttle2-FLAG-PI3KAP/XB130 was prepared as previously described (12), and pShuttle2-myc-PI3KAP/XB130 for expressing N-terminally myc-tagged PI3KAP/XB130 was constructed by myc-tagged PI3KAP/XB130 into the pShuttle2 vector. pShuttle2 plasmids for expressing FLAG-tagged or myc-tagged PI3KAP/XB130 deletion mutants were constructed by cloning each deletion mutant into the pShuttle2 vector. pEGFP plasmids for expressing GFP-fused PI3KAP/XB130 or its deletion mutants were constructed by cloning each fragment into the pEGFP-C1 vector (Clontech, Mountain View, CA, USA). pGEX vectors (GE Healthcare, Bukcinghamshire, UK) were used for expression of fusion proteins with GST in *Escherichia coli*. pGEX plasmids for expressing GST-fused PI3KAP/XB130 deletion mutants were constructed by cloning each deletion mutant into pGEX vectors.

### Transfection of Plasmids and siRNA

Transfection of plasmids into NIH3T3 cells or HEK293 cells was performed using Lipofectamine 2000 (NIH3T3 cells) or Lipofectamine LTX (HEK293 cells) according to the manufacturer’s protocol (Invitrogen). Transfection into HEK293T cells was carried out using a calcium phosphate precipitation method as previously described (29). Transfection of siRNA into FRTL-5 cells was performed using Lipofectamine RNAiMax according to the manufacturer’s protocol (Invitrogen). Random control siRNA and PI3KAP/XB130-specific siRNA were purchased from RNAi Corp. (Tokyo, Japan). The sequences of the PI3KAP/XB130 siRNA were 5′-CGGUCAAGUCUUCCAUAAAAC-3′ (sense strand) and 5′-UUUAUGGAAGACUUGACCGGA-3′ (antisense strand).

### Immunofluorescence Analysis

NIH3T3 cells were transfected with myc-tagged PI3KAP/XB130 or deletion mutants and then cultured in 10% FBS/DMEM overnight. Cells were washed once with PBS, fixed with 4% paraformaldehyde in PBS (4% PFA/PBS) for 10 min, and permeabilized with 0.2% Triton X-100 in PBS for 5 min at room temperature. Cells were then washed with PBS and incubated with 3% bovine serum albumin (BSA) in PBS for 1 h at room temperature, and primary antibodies (anti-Myc 9E10, 1:200 dilution) were added for 1 h at room temperature. The samples were again washed with PBS, incubated with 40 μM phalloidin-TRITC, and a secondary antibody conjugated to Alexa Fluor 488 (1:1000 dilution) for 1 h. The coverslips were washed again with PBS three times and then mounted in Vectashield for visualization using a confocal microscope Olympus FV500 (Olympus, Tokyo, Japan).

### Purification of GST Fusion Proteins

GST fusion proteins were prepared as described before (30). Briefly, pGEX plasmid was transformed into *E. coli* BL21 (DE3) pLysS. Expression of GST fusion proteins was induced by 1 mM Isopropyl β-d-thiogalactopyranoside (IPTG) overnight at 26°C. Cells were harvested and lysed by sonication three times for 30 s on ice in PBS containing 1% Triton X-100, 100 kallikrein-inactivating (KI) U/ml aprotinin, 20 μg/ml phenylmethylsulfonyl fluoride (PMSF), 10 μg/ml leupeptin, and 5 μg/ml pepstatin. The lysates were centrifuged, and supernatant was added to the Glutathione–Sepharose column (GE Healthcare). After washing with PBS, the GST fusion proteins were eluted by elution buffer (50 mM Tris–HCl, pH 8.0, and 10 mM reduced glutathione). The eluates were subjected to protein assay using a protein assay kit (Bio-Rad, Hercules, CA, USA).

### Purification of FLAG-Tagged Proteins

HEK293T cells were transfected with pShuttle2 plasmids coding for FLAG-tagged PI3KAP/XB130 or its deletion mutants. Cells were cultured for 2 days and then lysed at 0°C in 500 μl lysis buffer containing 50 mM Tris–HCl (pH 7.4), 150 mM NaCl, 1 mM NaF, 1 mM EDTA, 1 mM EGTA, 1% Triton X-100, 10% glycerol, 500 μM Na_3_VO_4_, 100 KI U/ml aprotinin, 20 μg/ml PMSF, 10 μg/ml leupeptin, and 5 μg/ml pepstatin. The lysates were centrifuged at 15,000 × *g* for 10 min at 4°C. The protein assay of the supernatant was performed using a protein assay kit (Bio-Rad). The cell lysates containing approximately 60 mg of protein were subjected to immunoprecipitation with anti-FLAG M2 antibody-conjugated agarose beads (Sigma-Aldrich). The immunoprecipitated FLAG-tagged proteins were eluted with FLAG peptide (Sigma-Aldrich). Concentrations of the FLAG-tagged proteins were determined by SDS-PAGE followed by coomassie brilliant blue (CBB) staining using serially diluted BSA as a standard.

### Blue Native-PAGE

Blue native (BN)-PAGE analysis was performed as previously described (31) with slight modifications. Briefly, the FLAG-tagged PI3KAP/XB130 protein was prepared using anti-FLAG antibody as described above, and then, the protein samples were mixed with 1/20 volume of 5% CBB G-250. The samples were separated by NativePAGE Novex Bis-Tris Gels (Invitrogen) according to the manufacturer’s protocols.

### Immunoprecipitation and Immunoblotting

Immunoprecipitation and Immunoblotting were performed according to standard procedures as described before (12). For immunoprecipitation of FLAG-tagged proteins, anti-FLAG M2 antibody-conjugated agarose beads were used.

### Actin Filament Pelleting Assay

Actin filament pelleting assay was carried out as previously described (32) with minor modifications. Actin monomers purified from rabbit skeletal muscle (AKL99) were purchased from Cytoskeleton, Inc. (Denver, CO, USA). F-actin was prepared by polymerizing actin monomers in F-buffer (50 mM KCl, 2 mM MgCl_2_, 0.2 mM ATP, and 0.2 mM DTT in 2M imidazole, pH 7.1) at room temperature for 30 min. Then F-actin (5 μM) was incubated with GST-fused PI3KAP/XB130 deletion mutants (2.5 μM) for 30 min at room temperature. After ultracentrifugation at 100,000 × *g* for 30 min at 4°C, the proteins in the supernatant or pellet fractions were analyzed by SDS-PAGE followed by CBB staining.

### Actin Crosslinking/Bundling Assay

Bovine serum albumin, FLAG-PI3KAP/XB130, or its deletion mutants (1 μM) were incubated with F-actin (5 μM) for 30 min at room temperature. Then the mixture was centrifuged at 8,000 × *g* for 20 min at 4°C. The supernatant or pellet fractions were analyzed by SDS-PAGE followed by CBB staining.

### Dextran Uptake Assay

HEK 293 cells seeded on coverslips were transfected with GFP-fused PI3KAP/XB130 or deletion mutants. Two days after transfection, the medium was replaced with 10% FBS/DMEM containing 0.2 mg/ml dextran–Texas Red 70,000 MW (Thermo Fisher Scientific, Lafayette, CO, USA) and then cells were incubated at 37°C for 4 h. In the case for FRTL-5 cells, cells grown on coverslips were transfected with siRNA and cultured for 24 h. The cells were serum-starved for 24 h in Coon’s F-12 medium (Sigma-Aldrich) containing 0.1% bovine serum albumin (BSA/F-12 medium). Then the medium was replaced with BSA/F-12 medium containing 1 nM TSH. After treatment with TSH for 16 h, dextran–Texas Red was added at a final concentration of 0.2 mg/ml, and then, the cells were incubated for 8 h (TSH treatment time was 24 h in total). After incubation with dextran–Texas Red, HEK293 cells and FRTL-5 cells were washed three times with PBS and fixed with 4% PFA/PBS for 10 min. After washing with PBS again, cells were stained with DAPI, and the coverslips were mounted in Vectashield for visualization using a confocal microscope. Immunofluorescence intensities of Texas Red were quantified and output as mean gray values using the ImageJ version 1.49 program (http://rsb.info.nih.gov/ij/; National Institutes of Health, Bethesda, MD, USA). The intensities were measured in individual GFP-positive cells (HEK293 cells) or in individual colonies (FRTL-5 cells).

### Statistical Analyses

Statistical analyses of data were performed by Student’s *t*-test or by one-way factorial ANOVA followed by Steel–Dwass *post hoc* multiple comparison test, as appropriate. *p* < 0.05 was considered statistically significant.

## Results

### PI3KAP/XB130 Was Colocalized with Actin Filaments through Its C-Terminal Region in Cells

**Figure 1:**
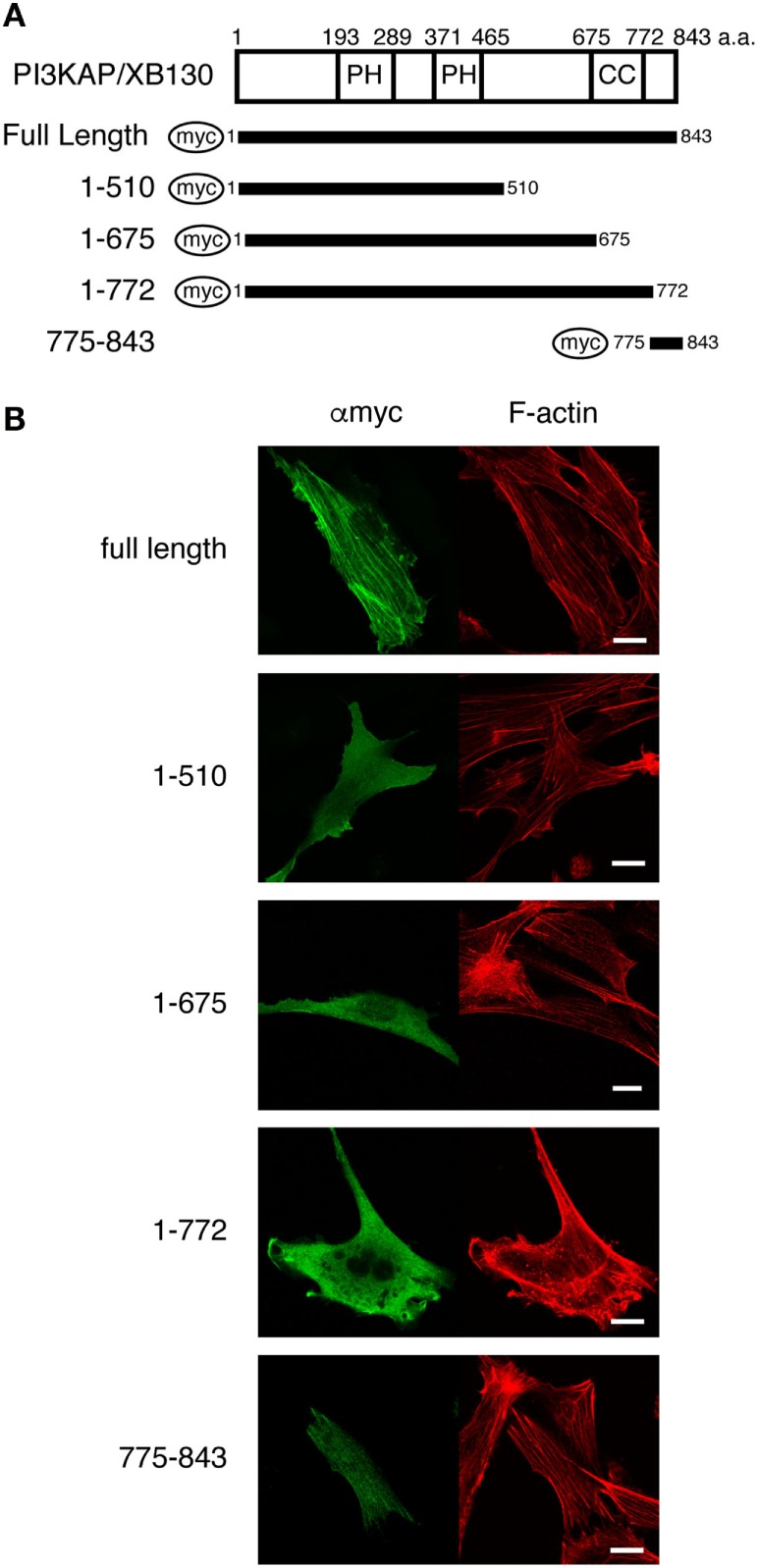
**PI3KAP/XB130 is localized to actin filaments**. **(A)** The myc-tagged PI3KAP/XB130 deletion constructs used in the experiments are shown. The numbers indicate the amino acid residues from the N-terminal end of PI3KAP. PH, PH domain; CC, coiled-coil domain. **(B)** Myc-tagged PI3KAP/XB130 or deletion mutants were transfected into NIH3T3 cells. Fixed and permeabilized cells were stained with anti-myc antibody and TRITC-labeled phalloidin. Myc-tagged proteins were visualized by Alexa488 in green in the confocal microscopy. F-actin was visualized in red.

At first, we searched for putative actin-binding region(s) by analyzing colocalization of several PI3KAP/XB130 deletion mutants (Figure 1A) with actin filaments. We transfected NIH3T3 cells with myc-tagged PI3KAP/XB130 and found that PI3KAP/XB130 was localized to actin stress fibers in NIH3T3 cells (Figure 1B). We further investigated localization of PI3KAP/XB130 deletion mutants to stress fibers. As shown in Figure 1B, deletion mutants, in which C-terminal regions were deleted (1-510, 1-657, and 1-772), were diffused in the cytosol, and their localization did not correspond to actin filaments. These results indicated that a C-terminal sequence of approximately 70 amino acids was required for localization to actin filaments. Consistently with these results, a deletion mutant consisting of C-terminal 69 amino acid residues (775–843) showed filamentous patterns corresponding to actin filaments (Figure 1B). These results showed that a region responsible for PI3KAP/XB130 colocalization with actin filaments existed within its C-terminal region 775-843.

### PI3KAP/XB130 Bound to Actin Filaments through Its C-Terminal Region *In Vitro*

**Figure 2:**
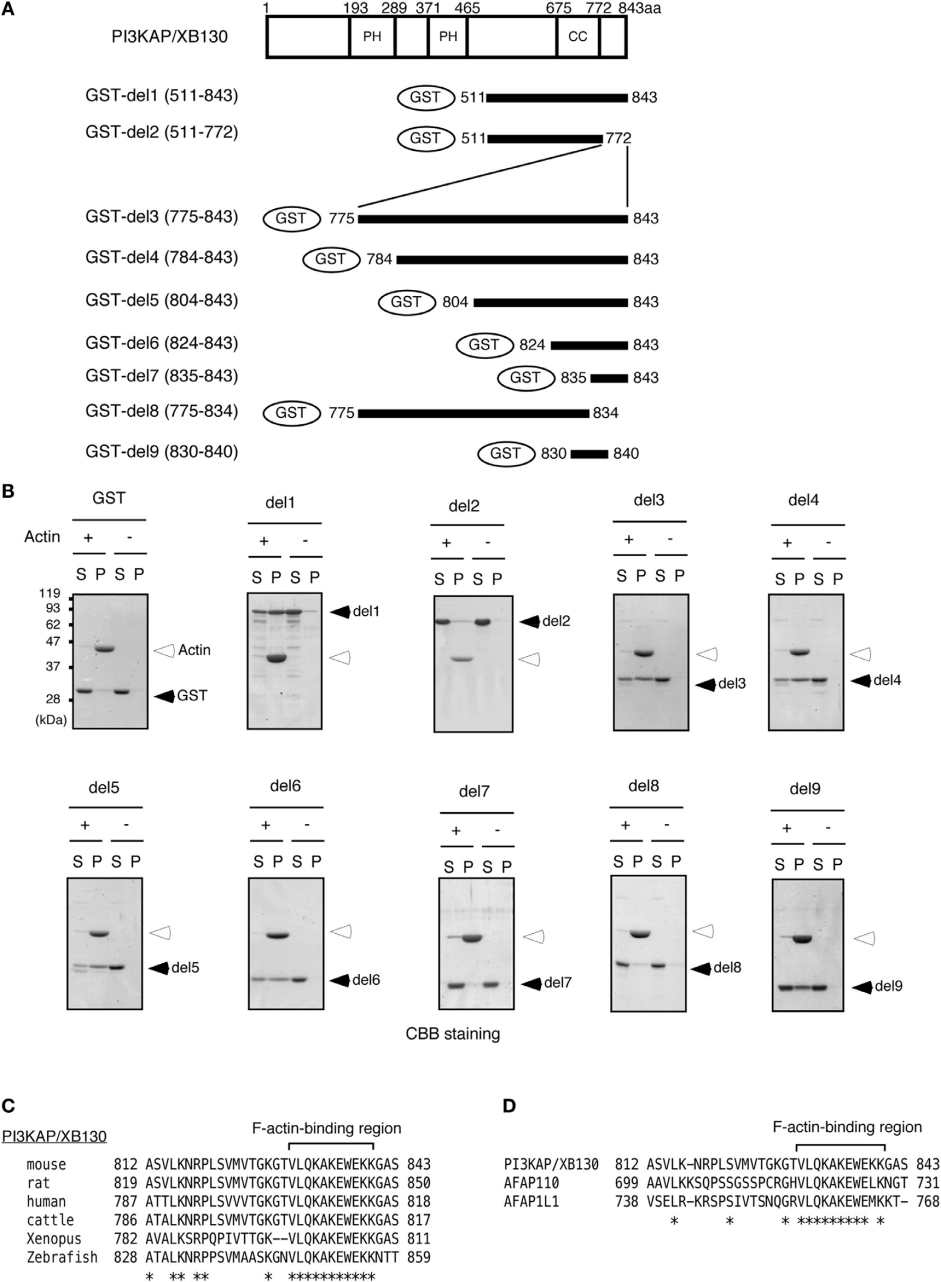
**PI3KAP/XB130 binds to actin filaments**. **(A)** The GST-fused PI3KAP/XB130 deletion constructs used in the experiments are shown. The numbers indicate the amino acid residues from the N-terminal end of PI3KAP/XB130. PH, PH domain; CC, coiled-coil domain. **(B)** Actin filaments polymerized *in vitro* were incubated with GST or GST-fused PI3KAP/XB130 deletion mutants. The mixture of actin filaments and GST-fusion proteins was subjected to ultracentrifugation, and the fractions of supernatant (S) and pellet (P) were separated by SDS-PAGE and then proteins were visualized by CBB staining. **(C,D)** Alignment of amino acid sequences of F-actin-binding region of PI3KAP/XB130 among mouse, rat, human, cattle, frog, and zebrafish **(C)** or among mouse PI3KAP/XB130, AFAP-110, and AFP1L1 **(D)**. The asterisks indicate identical amino acid residues.

Colocalization of PI3KAP/XB130 with actin filaments prompted us to examine whether PI3KAP/XB130 can directly bind to actin filaments. To address this question, we performed an actin filament pelleting assay using recombinant PI3KAP/XB130 fragments fused to GST (Figure 2A). In this *in vitro* assay, fragments that can bind to actin filaments will co-sediment with actin filaments by ultracentrifugation, but fragments that cannot bind will not. In control samples without actin, GST or GST-fused PI3KAP/XB130 fragments did not sediment by ultracentrifugation (Figure 2B), indicating that these recombinant proteins did not self-aggregate to form pellets. When incubated with actin, a C-terminal fragment of PI3KAP/XB130 (amino acid residues 511–843; GST-del1) cosedimented with actin filaments, while GST alone did not (Figure 2B), indicating that GST-del1 bound to actin filaments *in vitro*. In addition, deletion of the C-terminal 70 amino acids from GST-del1 (GST-del2) abolished the cosedimentation, and these 70 amino acid residues (GST-del3) cosedimented with actin filaments. To further analyze minimal regions responsible for actin binding, we performed the pelleting assay using shorter PI3KAP/XB130 fragments. As shown in Figure 2B, PI3KAP/XB130 fragments containing amino acid residues 830–840 (GST-del4, 5, 6, and 9) cosedimented with actin filaments. However, when this region was separated into two regions (GST-del7 and 8), cosedimentation of these fragments was not observed. These results indicated that PI3KAP/XB130 bound to actin filaments through amino acid residues 830–840 and that this region needed to be intact. Furthermore, homology search analysis revealed that this region was highly conserved in many vertebrate species (Figure 2C). This region was also conserved in the C-terminal region of other AFAP family members (Figure 2D), which was different from the known ABD in AFAP-110 and AFAP1L1.

### PI3KAP/XB130 Crosslinked Actin Filaments *In Vitro*

**Figure 3:**
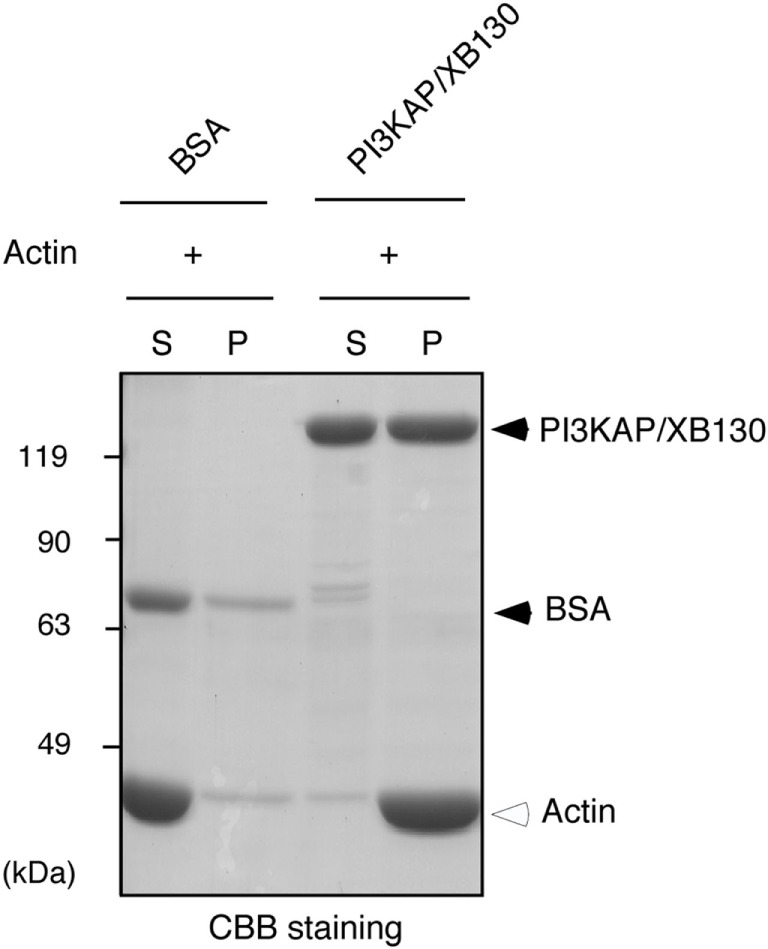
**PI3KAP/XB130 crosslinks actin filaments**. Actin filaments polymerized *in vitro* were incubated with BSA or FLAG-tagged recombinant PI3KAP/XB130. The mixture was subjected to low-speed centrifugation, and the fractions of supernatant (S) and pellet (P) were separated by SDS-PAGE and then proteins were visualized by CBB staining.

Some actin-binding proteins are known to crosslink actin filaments to form actin meshwork or bundles. Therefore, we next tested whether PI3KAP/XB130 could crosslink actin filaments. To examine actin-crosslinking ability of PI3KAP/XB130, we performed an *in vitro* crosslinking assay. In this assay, actin filaments are pelleted by low-speed centrifugation when they are mixed with an actin-crosslinking protein, although actin filaments alone remain in the supernatant. As shown in Figure 3, when actin was mixed with negative control BSA, actin remained in the supernatant fraction. By contrast, when actin was mixed with recombinant full-length PI3KAP/XB130, actin was detected in the pellet fraction. These results indicated that PI3KAP/XB130 could crosslink actin filaments *in vitro*.

### PI3KAP/XB130 Formed Multimers through Its N-Terminal Region

**Figure 4:**
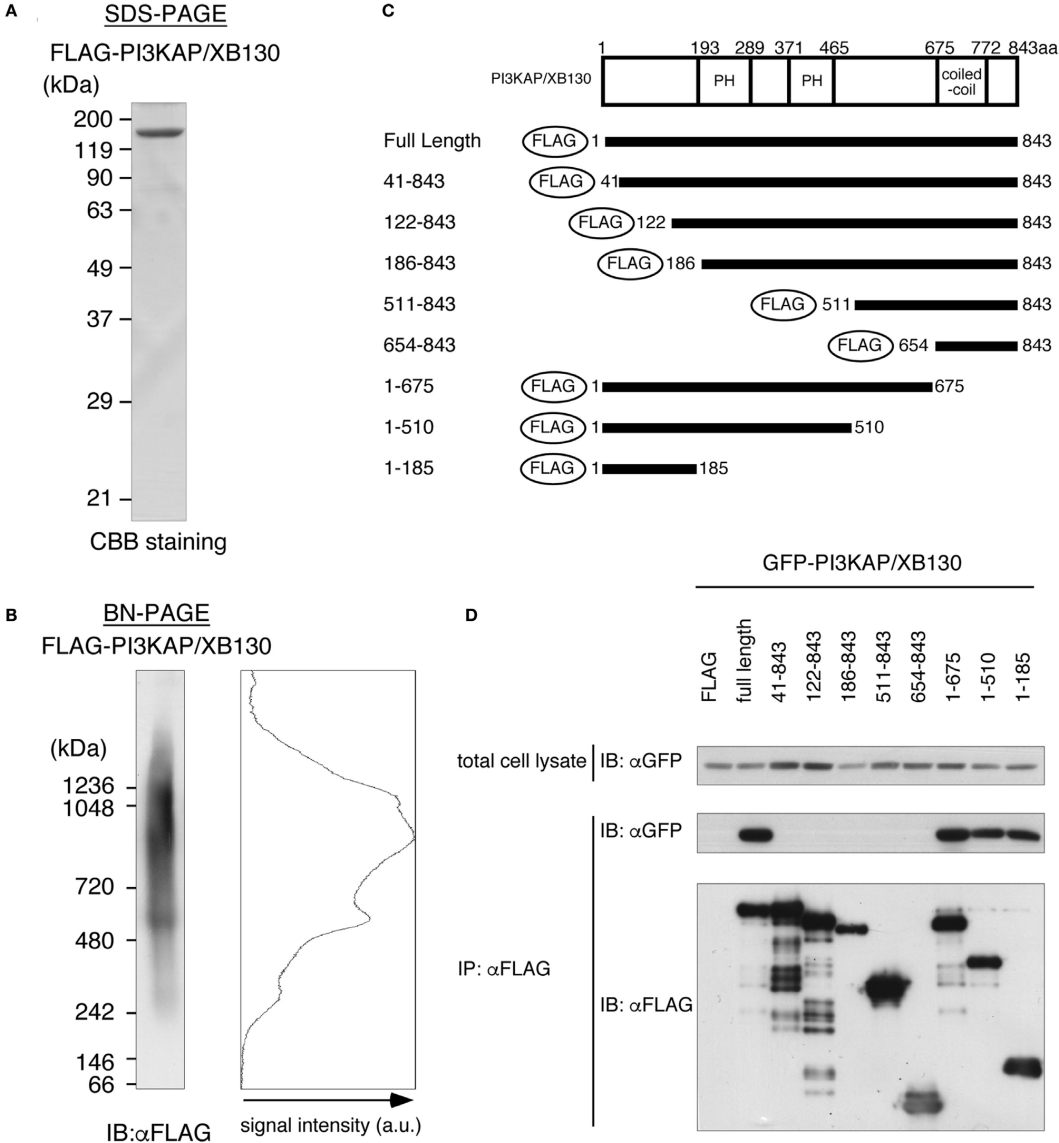
**PI3KAP/XB130 forms a multimer**. **(A,B)** FLAG-tagged recombinant PI3KAP/XB130 was purified from HEK293T cells and then subjected to SDS-PAGE **(A)** or BN-PAGE **(B)**. **(C)** FLAG-tagged PI3KAP/XB130 deletion constructs used in the co-immunoprecipitation assays are shown. The numbers indicate the amino acid residues from the N-terminal end of PI3KAP/XB130. PH, PH domain; CC, coiled-coil domain. **(D)** GFP-PI3KAP/XB130 was co-transfected with full length or deletion mutant of FLAG-tagged PI3KAP/XB130 into HEK293T cells. The cell lysates were immunoprecipitated with anti-FLAG antibody, and then, the samples were subjected to immunoblotting with indicated antibodies.

In general, actin-crosslinking proteins that have one actin-binding site within a molecule need to form a dimer or a multimer to crosslink actin filaments. Then, to investigate dimerization/multimerization of PI3KAP/XB130, we analyzed electrophoretic mobilities of recombinant PI3KAP/XB130 under native conditions that maintain protein complexes using BN-PAGE. FLAG-PI3KAP/XB130 was immuno-purified from HEK293T cells. Endogenous expression of PI3KAP/XB130 was not detected in HEK293T cells (data not shown). The recombinant FLAG-PI3KAP/XB130 was subjected to SDS-PAGE followed by CBB staining to confirm its purity. We did not detect any protein other than FLAG-PI3KAP/XB130 at a CBB staining level (Figure 4A). The immuno-purified FLAG-PI3KAP/XB130 was subjected to BN-PAGE followed by immunoblotting using anti-FLAG antibody. PI3KAP/XB130 was detected at approximately 250 kDa, 500 kDa, and 900–1200 kDa in BN-PAGE (Figure 4B), even though PI3KAP/XB130 is usually detected at approximately 125 kDa in SDS-PAGE (Figure 4A). These results suggest that PI3KAP/XB130 formed multimers. To further confirm the multimerization, we co-transfected GFP-PI3KAP/XB130 with FLAG-PI3KAP/XB130 into HEK293T cells and then analyzed their binding by co-immunoprecipitation assays. As shown in Figure 4D, GFP-PI3KAP/XB130 was co-immunoprecipitated with FLAG-PI3KAPXB130, indicating that PI3KAPXB130 bound to itself in cells. Furthermore, we searched for region(s) necessary for the multimerization using several deletion mutants of PI3KAP/XB130 (Figure 4C). FLAG-tagged deletion mutants that lack at least 40 amino acids in the N-terminus (amino acid residues 41–843, 122–843, 186–843, 511–843, and 654–843) did not bind to GFP-PI3KAP/XB130, and deletion mutants containing N-terminal region (amino acid residues 1–675, 1–510, and 1–185) bound to it (Figure 4D). These results indicate that PI3KAP/XB130 formed multimers through its N-terminal region.

### The Multimerizing Region and Actin-Binding Region Were Required for PI3KAP/XB130 to Crosslink Actin Filaments

**Figure 5:**
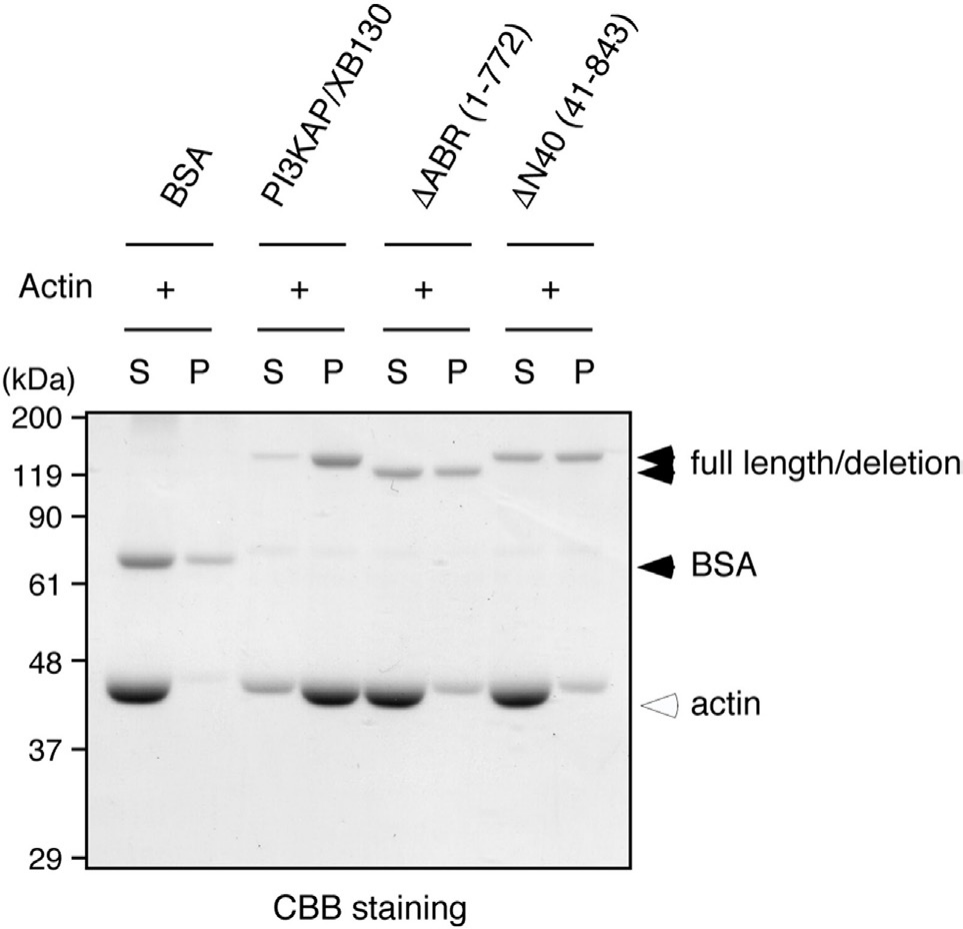
**Actin crosslinking by PI3KAP/XB130 requires the dimerizing region and actin-binding region**. Actin filaments polymerized *in vitro* were incubated with BSA, full-length PI3KAP/XB130 or the deletion mutants (ΔABR or ΔN40). The mixture was subjected to low-speed centrifugation, and the fractions of supernatant (S) and pellet (P) were separated by SDS-PAGE and then proteins were visualized by CBB staining.

We next examined whether multimerization and actin binding of PI3KAP/XB130 could contribute to the actin crosslinking ability. We carried out the *in vitro* crosslinking assay using PI3KAP/XB130 deletion mutants that lack the C-terminal actin-binding region (ΔABR) or the N-terminal 40 amino acids (ΔN40). We found that full-length PI3KAP/XB130 crosslinked actin filaments, but neither ΔABR nor ΔN40 showed the crosslinking ability (Figure 5). These results indicate that both of the C-terminal actin-binding region and the N-terminal multimerizing region were required for PI3KAP/XB130 to crosslink actin filaments.

### PI3KAP/XB130 Enhances Endocytosis through the Actin-Binding Region At Least in a Part

**Figure 6:**
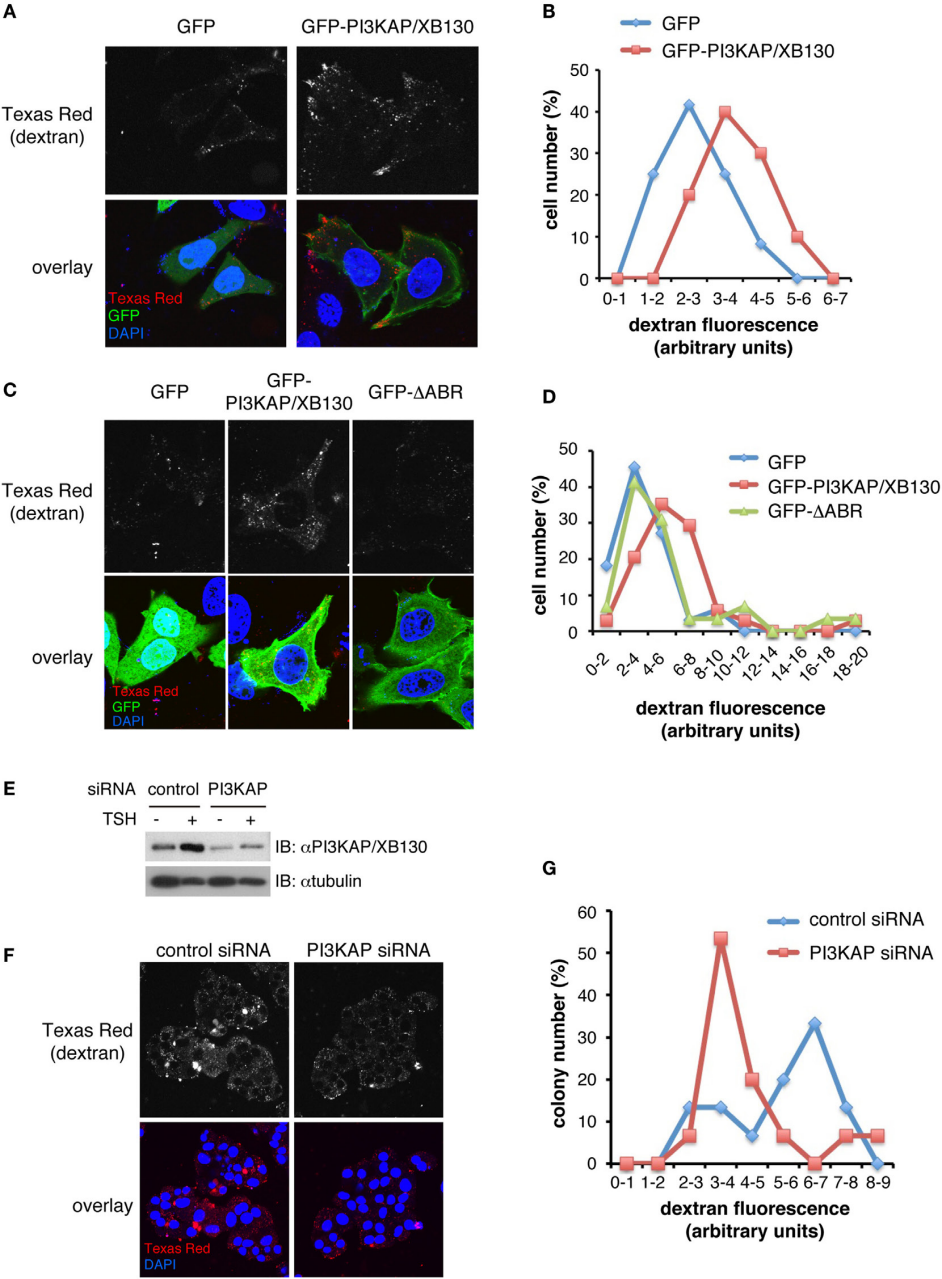
**PI3KAP/XB130 promotes dextran endocytosis through the actin-binding region**. HEK293 cells were transfected with GFP, GFP-PI3KAP, or GFP-ΔABR in the presense of 10% FBS for 48 h **(A–D)**. FRTL-5 cells were transfected with control or PI3KAP/XB130-specific siRNA, serum-starved for 24 h, and treated with 1 nM TSH for 24 h **(E–G)**. The cells were incubated with 0.2 mg/ml dextran–Texas Red during the last 4 h [HEK293 cells; **(A–D)**] or 8 h [FRTL-5 cells; **(F,G)**]. After fixation and DAPI staining, dextran–Texas Red incorporated into cells was visualized by confocal microscopy. **(A,C,F)** Representative images of cells are shown. Gray scale images of Texas Red are indicated in upper panels and overlay images in lower panels (red, Texas Red; green, GFP; and blue, DAPI). **(B,D,G)** Amounts of dextran–Texas Red incorporated into cells were evaluated by the ImageJ software. The percentage of cells or colonies in each fraction of fluorescent intensity was indicated in histograms. The fluorescent values were analyzed by Student’s *t*-test [**(B)** *n* = 10–12, **(G)** *n* = 15] or by ANOVA followed by Steel–Dwass *post hoc* test [**(D)** *n* = 30–34]. **(E)** PI3KAP/XB130 protein levels were analyzed by immunoblotting.

We further investigated a role of PI3KAP/XB130 in endocytosis, an actin-related process. To examine this, we analyzed incorporation of fluorescence-labeled dextran as an index of endocytosis in HEK293 cells. As shown in Figure 6A, Texas Red-labeled dextran incorporated into cells was detected as small dots in GFP-transfected control cells and GFP-PI3KAP/XB130-transfected cells (Figure 6A). To analyze dextran incorporation more quantitatively, we measured fluorescent intensities of Texas Red in GFP-positive cells (Figure 6B). This analysis revealed that the Texas Red intensities of the most abundant population were higher in GFP-PI3KAP/XB130-transfected cells than in GFP-transfected cells, and the difference of values between these two groups was statistically significant (Student’s *t*-test, *p* < 0.01). These results suggest that GFP-PI3KAP/XB130 enhanced endocytosis. To further analyze effects of the ΔABR mutant, which lacks actin-binding region, we transfected cells with GFP-ΔABR and quantified amounts of incorporated dextran (Figures 6C,D). Unlike the case for GFP-PI3KAP/XB130, which enhanced dextran uptake, most of the cells expressing GFP-ΔABR exhibited similar dextran uptake as GFP-expressing control cells. In addition, small number of cells showed relatively high fluorescent values of Texas Red (>10) even in GFP-ΔABR-transfected cells. Statistical analysis showed that the difference of the Texas Red values between GFP and GFP-PI3KAP/XB130 was statistically significant (ANOVA followed by Steel–Dwass *post hoc* test, *p* < 0.05), and there were no statistically significant differences between GFP and GFP-ΔABR or between GFP-PI3KAP/XB130 and GFP-ΔABR. These data suggest that, for PI3KAP/XB130 to control endocytosis properly, at least the actin-binding region is required. To examine a role of PI3KAP/XB130 in endocrine cells, we knocked down expression of this protein in a thyroid cell line FRTL-5 using siRNA and tested effects on dextran uptake. As previously described (12), PI3KAP/XB130 protein levels were increased in TSH-treated cells, and this effect was inhibited by transfection of siRNA against PI3KAP/XB130 (Figure 6E). The dextran uptake assay showed a tendency for dextran uptake to be inhibited in PI3KAP/XB130 knockdown cells (Figures 6F,G), but statistically, there were not significant differences between control cells and knockdown cells (Student’s *t*-test, *p* = 0.11).

## Discussion

Actin filaments exist in cells as single filaments or as meshworks or bundles in which actin filaments are crosslinked by many kinds of actin-binding proteins. Together with initiation of *de novo* actin polymerization, these crosslinked structures are required to realize the diversity of actin filament structures, leading to induction of cell migration, maintenance of cell shapes, endocytosis of macromolecules, and transport of intracellular vesicles (7, 33). Therefore, it is obvious that elucidating functions of actin-crosslinking proteins is important to understand these actin-related biological processes. In this study, we demonstrated that PI3KAP/XB130 was a novel actin-crosslinking protein and characterized molecular mechanisms for PI3KAP/XB130-mediated actin crosslinking. We found that the C-terminal region of PI3KAP/XB130 directly bound to actin filaments (Figure 2), and that PI3KAP/XB130 formed multimers *via* its N-terminal region (Figure 4). Furthermore, deletion mutants lacking the C-terminal region or the N-terminal region did not crosslink actin filaments (Figure 5). These results suggest that PI3KAP/XB130, which multimerized through its N-terminal region, binds to actin filaments through its C-terminal region and then crosslinks the filaments.

Actin-binding domain of AFAP family protein was originally discovered in AFAP-110 (13). Among AFAP family members, this ABD is conserved in AFAP1L1. It has been reported that AFAP1L1 is also localized to actin filaments, suggesting that this ABD functions in AFAP1L1 as well (10). However, this ABD is not conserved in PI3KAP/XB130. Nevertheless, PI3KAP/XB130 was localized to actin stress fibers (Figure 1). In contrast to these results, we and others have reported that PI3KAP/XB130 was localized to lamellipodia in thyroid carcinoma cells (16) and to lamellipodia-like cortical actin structures in non-transformed thyroid follicular cells (12), suggesting that intracellular localization of this protein was not restricted to lamellipodia but was dependent on actin filament structures. These observations led us to speculate that PI3KAP/XB130 might possess a novel ABD. At first, we searched for a putative ABD in PI3KAP/XB130 by analyzing colocalization of PI3KAP/XB130 deletion mutants with actin stress fibers and found that the C-terminal region of PI3KAP/XB130 was necessary and sufficent to be localized to actin stress fibers. Lodyga et al. also reported that this carboxyl terminal region was required for localization to lamellipodial actin meshwork (16), supporting that this region was important for association of PI3KAP/XB130 with actin filaments. Therefore, we performed biochemical binding assays using C-terminal fragment of PI3KAP/XB130 and demonstrated that the C-terminal 10-amino acid sequence of PI3KAP/XB130 bound to actin filaments and this sequence was distinct from AFAP-110 ABD. Moreover, we could not find any similarity between this 10-amino acid sequence and other known ABDs, suggesting that this region functions as a novel ABD. Interestingly, this 10-amino acid sequence of PI3KAP/XB130 was conserved in the other AFAP family members, AFAP-110 and AFAP1L1 (Figure 2), indicating that this actin-binding region may contribute to actin binding of these proteins.

In addition to the C-terminal actin-binding domain in PI3KAP/XB130, Lodyga et al. reported that N-terminal region of PI3KAP/XB130 was also localized to lamellipodial actin meshwork using a human construct of PI3KAP/XB130 (16). By contrast, we did not find any colocalization with N-terminal region of PI3KAP/XB130 using a mouse construct, suggesting species-specific mechanisms in localization to actin filaments. Lodyga et al. speculated that an actin-binding K/RYKXL motif, which resides in the N-terminal segment of human PI3KAP/XB130, might contribute to the localization by searching for actin-binding motifs using amino acid sequences. However, this motif is not conserved in mouse PI3KAP/XB130 that we used in this study (variant 1; GenBank Accession # NM_001177796), because the leucine residue within the motif is replaced with glutamine in the mouse sequence. Interestingly, there also exists a splice variant (variant 2; GenBank Accession # NM_001177797) that lacks the second exon in mouse PI3KAP/XB130 variant 1, and this alternative splicing yields the K/RYKXL motif in the N-terminus of the isoform produced from this splice variant. These differences in the actin-binding motif sequence in N-terminus suggest differential regulatory mechanisms for localization to actin filaments among splice variants in PI3KAP/XB130, and the differential roles of these variants need to be further elucidated.

We further analyzed possible mechanisms for PI3KAP/XB130 to regulate actin cytoskeletons and found that PI3KAP/XB130 crosslinked actin filaments. Moreover, we also found that PI3KAP/XB130 formed a multimer. These results suggested that PI3KAP/XB130, which had one actin-binding site, crosslinked actin filaments by making at least two actin-binding sites through multimerization. Our results that a multimerization-deficient mutant ΔN40 did not crosslink actin filaments strongly supported this hypothesis. AFAP-110 has also been reported to form a multimer and crosslink actin filaments (14, 34). Therefore, in terms of actin crosslinking, the function is likely to be conserved among AFAP family proteins. However, molecular mechanisms underlying the actin crosslinking appear to be different. Self-association of AFAP-110 is mediated by at least two regions of AFAP-110, its PH1 domain and C-terminal LZIP motif (35). These observations are different from PI3KAP/XB130, which requires N-terminal 40 amino acids for multimerization and actin-crosslinking, as described above. Because this 40-amino-acid region does not contain apparent domains or motifs that function in protein–protein interaction, the multimerization of PI3KAP/XB130 may occur through a novel mechanism.

In regard to the actin cytoskeleton, PI3KAP/XB130 has been reported to play an important role in lamellipodia formation and cell migration (16, 17, 36), but there were no reports showing its involvement in other biological processes related to actin. At first, we tried to demonstrate the involvement of the actin-binding ability of PI3KAP/XB130 in cell proliferation induced by TSH and IGF-I, because PI3KAP/XB130 is important for the signaling crosstalk as we previously reported (12). However, our preliminary data indicated that the effects of the ΔABR mutant were similar to wild-type PI3KAP/XB130 (unpublished data), suggesting that the actin-binding ability was not involved in the regulation of cell proliferation. Therefore, for the purpose of finding a novel process in which PI3KAP/XB130 is involved, we next verified effects of overexpression of this protein on endocytosis. Endocytosis is important for uptake of nutrients, regulation of signal transduction, and control of osmotic pressure, and the actin cytoskeleton plays an indispensable role in these steps (37). In the present study, we demonstrated, for the first time, that PI3KAP/XB130 overexpression enhanced endocytosis using fluorescence-labeled dextran (Figure 6). We further performed functional studies using PI3KAP/XB130 deletion mutants and found that most of the cells transfected with the ΔABR mutant lacking the actin-binding region showed dextran uptake similar to GFP-expressing control cells, suggesting that the actin-binding region is required for proper control of endocytosis by PI3KAP/XB130. However, we also observed that small number of cells incorporated high levels of dextran in ΔABR-expressing cells, and this fact indicates that PI3KAP/XB130 could enhance endocytosis through mechanisms independent of its actin-binding ability. PI3KAP/XB130 have several motifs and domains that can function in signal transduction, protein–protein interaction, or lipid-binding (11, 12, 23), and these motifs and domains may be involved in the mechanisms. In addition, loss-of-function analysis in a thyroid cell line raised a possibility that PI3KAP/XB130 is necessary for the regulation of endocytosis in endocrine cells (Figure 6). It is interesting that, in the thyroid, endocytic pathways play an important role in the control of thyroid hormone release (2), and further studies are necessary to uncover the relevance of PI3KAP/XB130-mediated endocytosis to endocrine cell physiology. Meanwhile, in cancer cells, endocytic pathways are important not only for the non-selective uptake of nutrients including amino acids (5) but also for cell migration and invasion through multiple ways such as integrin trafficking and matrix metalloprotease transport (6). Thus, PI3KAP/XB130-mediated endocytosis may be a clue to understand these processes in endocrine cancer cells.

In summary, our data clearly demonstrated that PI3KAP/XB130 crosslinked actin filaments. Furthermore, its ability to crosslink actin filaments required both the N-terminal multimerizing region and the C-terminal novel actin-binding region. Taken together with the results that PI3KAP/XB130 has positive effects on endocytosis, we propose that PI3KAP/XB130 is a novel actin crosslinking protein and plays an important role in endocytosis.

## Author Contributions

DY, TA, KC, SM, KI, FH, and S-IT designed research. DY, TA, FH, and S-IT acquired, analyzed, and interpreted data. DY, FH, and S-IT drafted this paper and TA, KC, SM, and KI revised it. All authors approved the final manuscript and agreed to be accountable for all aspects of this work.

## Conflict of Interest Statement

The authors declare that the research was conducted in the absence of any commercial or financial relationships that could be construed as a potential conflict of interest.
